# Assessing Africa’s position in the development of AI-enabled ECG devices

**DOI:** 10.12688/f1000research.154316.2

**Published:** 2025-08-19

**Authors:** Hamza Ameziane, Yassine Zahidi, Mohamed El-Moufid, Hicham Medromi, Nadia Machkour, Nabila Rabbah

**Affiliations:** 1 Laboratory of Complex Cyber Physical Systems (LCCPS), Hassan II University of Casablanca National School of Arts and Crafts, Casablanca, Grand Casablanca, Morocco; 2 Foundation for Research Development and Innovation in Science and Engineering, Mohammedia, Morocco; 3 The International Academy of Scientific Francophonie (AIFS), Rabat, Morocco

**Keywords:** Artificial Intelligence (AI), cardiovascular disease, Cardiac Medicine, Electrocardiogram (ECG), Healthcare Technology, VOSviewer

## Abstract

**Background:**

The integration of Artificial Intelligence (AI) in electrocardiographic (ECG) devices has become a pivotal area of research, particularly during the COVID-19 pandemic. These technologies are essential for enhancing cardiac diagnosis and monitoring.

**Methods:**

This study assesses current trends, key contributors, and collaborative networks in the field of AI-enhanced ECG devices. We utilized a comprehensive analysis, using the Biblioshiny library from Bibliometrix for data exploration of data extracted from the Scopus database and VOSViewer for creating and visualizing maps. These tools were played an important role in conducting an in-depth analysis of the relationships and developments within the field.

**Results:**

The analysis shows a significant increase in publications related to AI-enhanced ECG devices, with a marked surge during the COVID-19 pandemic. Despite the growing interest and technological advancements, the study exposes a notable disparity in the geographical distribution of research contributions, highlighting substantial under-representation of African researchers. This gap is attributed to infrastructural, financial constraints, and limited collaborative networks within the continent.

**Conclusion:**

The rapid evolution and increasing importance of AI in ECG devices underscore the need for more inclusive research practices. There is a critical need to integrate and promote contributions from under-represented regions, particularly Africa, to ensure a globally diverse perspective in tackling health challenges. This study calls for enhanced participation and support for African researchers to bridge the existing research gap and foster global health equity.

## I. Introduction

The electrocardiogram (ECG) is a fundamental diagnostic device in cardiac medicine. It records cardiac activity using electrodes placed on the skin. The ECG is used to detect a multitude of cardiac pathologies, including arrhythmias, myocardial infarction and other cardiac disease. ^1^ The development of the ECG dates back to the late 19th century, with the pioneering work of Willem Einthoven, a Dutch physiologist. Einthoven invented the electrocardiograph in 1903, a device capable of recording the heart’s electrical signals. For his major contributions, he was awarded the Nobel Prize in Physiology or Medicine in 1924. ^2^ The ECG functions by measuring the variations in electrical potential produced by the heart during the depolarization and repolarization cycles of cardiac cells. These variations are picked up by electrodes placed at different points on the body. The resulting trace is called the electrocardiogram which is a graphical representation of the heart’s electrical activity. ^3^ He is used in a variety of clinical settings to diagnose and monitor cardiac conditions. For example, it is important in the detection of cardiac arrhythmias, which can be intermittent and require prolonged monitoring to be captured. Modern devices enable this continuous monitoring. This improves clinicians’ ability to diagnose and manage these conditions. ^4^ Innovations in sensor and wireless technologies have also made ECG data collection more comfortable and less intrusive for patients. This facilitates wider and more effective use of this technology. ^5– 7^ With the evolution of technology, ECG devices have become more portable and accessible. Recent innovations include wearable ECG machines and wearable devices such as smartwatches, which enable continuous, real-time monitoring. ^3, 8, 9^ The integration of artificial intelligence (AI) into ECG devices has enabled the development of more accurate and efficient detection algorithms, capable of processing and analyzing large amounts of data in real time. ^10– 12^ The new generation of ECGs regrouping lot of technologies like tele-ECG and bio-authentication in order to facilitate use and enhance performance. However, to improve ECG signal analysis, several optimization techniques have been used like Spectrogramming and optimization by spider monkeys. This enables better interpretation of the recorded data. ^13^ Supervised convolutional neural networks with contrastive learning have also shown significant improvement in the classification of ECG arrhythmias from multichannel signals. ^11– 13^

The aim of this study is to examine the global landscape of research on AI-based ECG devices, with a particular focus on Africa’s participation, visibility and collaborations. In contrast to existing work that focuses on technical or clinical aspects, this article offers a novel perspective that highlights geographical equity and structural gaps in scientific production. The originality of this study lies in the quantitative analysis of Africa’s position based on data from the Scopus database.

To achieve this, the present research focuses on scientific articles published over the last five years (2019-2024). It provides an overview of the evolution of research directions in the field of artificial intelligence applications in cardiology. This allows for the mapping of author networks, institutional output, and transnational collaborations, particularly in the context of emerging technologies in the field of ECG-based cardiac care.

The results of this research should inform international funding agencies, health policymakers, and AI developers about the efforts needed to ensure the equitable inclusion of low- and middle-income countries in future medical innovations involving AI.

The building of the study’s structure is based mainly on answering the following questions:

1. What are the main publication trends in the field of ECG devices?
2. Who are the most influential authors, institutions, and countries?
3. What collaborations exist between researchers and institutions?
4. What are the involving technologies and innovations applied in ECG devices?

By answering these questions, the study offers practical insights for:

• national and international health research funders;
• institutions aiming to build scientific leadership in Africa;
• and developers of ECG-AI technologies looking for global scalability and ethical deployment.

The paper is structured as follows, the first section is the methods used to collect data. The second section will be dedicated to description of the electrocardiogram. The third section is dedicated to the analysis and discussion of the results, the fourth section highlights the limitations of this study and the last section presents the conclusion.

## II. Methods

**Figure 1.**
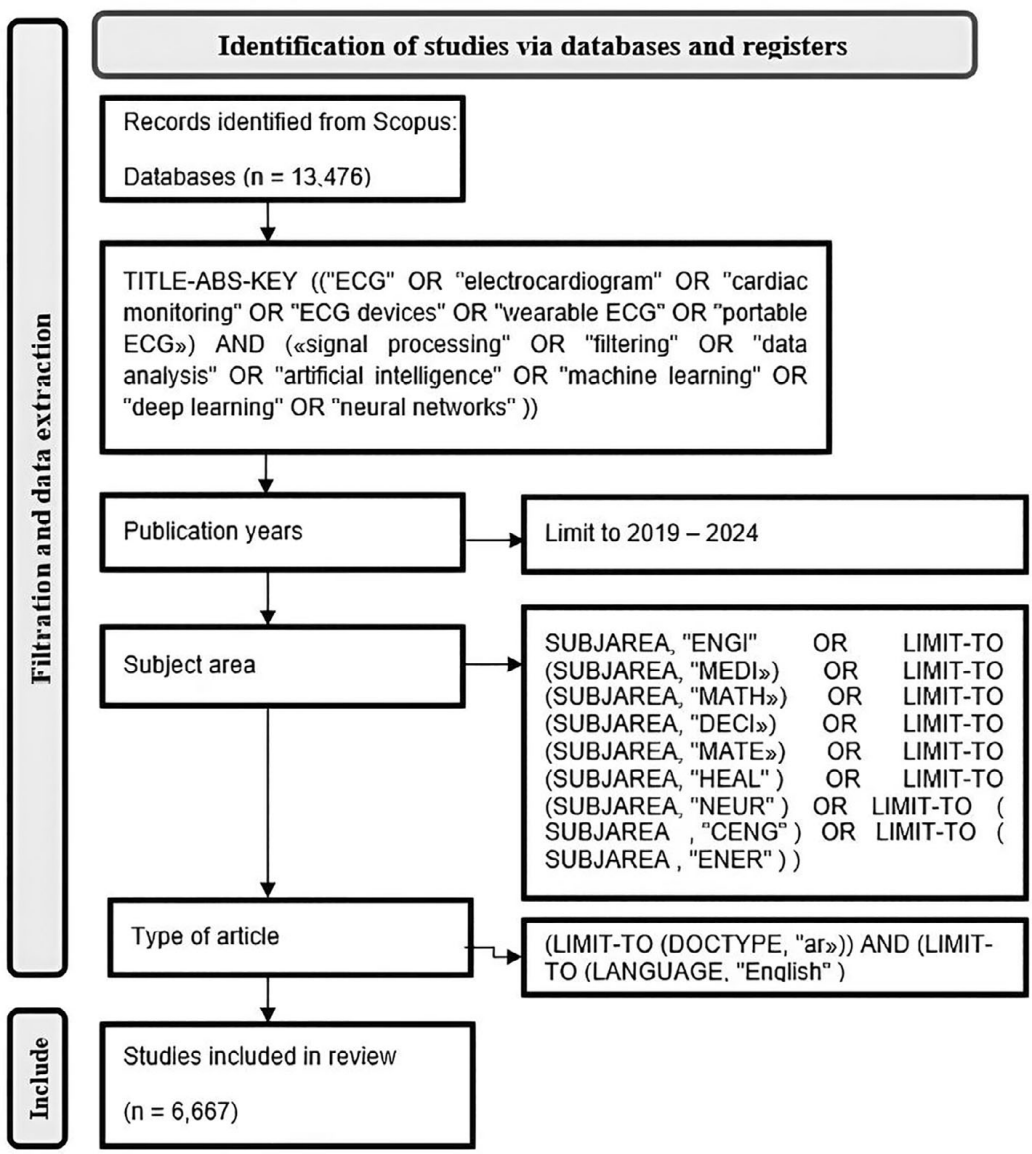
Summary of the data collection and screening.

The analysis is carried out using the Scopus database. A reliable database in terms of sources, managed by independent experts who are recognized leaders in their field. ^14, 15^ Figure 1 is a presentation of the data collection methodology used for this analyze on the application of artificial intelligence in electrocardiography. We performed exhaustive research of the Scopus database to identify relevant publications from the period 2019 to 2024. In the first time, we retrieved over than 24,000 records Using a combination between significant keywords like “ECG”, “electrocardiogram”, “cardiac monitoring”, “signal processing”, “data analysis”, “artificial intelligence” and “neural networks”. To ensure the relevance of our data, we imposed a number of filters. The publication period was limited to the last 5 years, in order to focus on recent advancements. We also limited the search to English-language documents to facilitate a consistent review process.

The final selection included 6,667 studies meeting our specific criteria, encompassing important aspects of AI integration in ECG technology. Each selected study was documented with detailed bibliographic information, including authors, title, abstract and keywords. This comprehensive dataset forms the basis of our subsequent analysis, aimed at effectively analyzing trends and contributions in the field.

To analyze this data, we used the Biblioshiny library from Bibliometrix. This tool has enabled us to explore bibliometric data in an interactive and intuitive way. In addition, we also used VOSViewer ^16^ to create and visualize bibliometric maps using the VOS mapping technique. These techniques based on developed mathematical algorithms helped us illustrate the relationships and connections between the different articles. All these tools have been essential for conducting a thorough and insightful data analysis, thus revealing major trends, key relationships and significant developments in the field studied.

To assess Africa’s specific contribution and performance in research, we used the following quantitative indicators:

○ Number of publications (total number of articles written by the authors),
○ Number of citations (total number and average number of citations per article),
○ MCP ratio (ratio of multi-country publications indicating international collaboration),
○ Institutional visibility (presence of African institutions in co-author and productivity rankings),
○ and h-index of lead authors affiliated with African institutions.

These indicators enabled us to measure the scientific visibility, intensity of collaboration, and impact levels of African countries relative to global benchmarks.

## III. Description of the electrocardiogram (ECG)

### A. Basic principle

The electrocardiogram (ECG) is an essential diagnostic device that records the heart’s electrical activity over time using electrodes positioned on the skin and precisely around the heart. It is widely used in medical practice to assess the condition of the heart, identify any irregularities and guide the treatment of various heart diseases. It provides a non-invasive, rapid and reliable method of monitoring heart rhythm and detecting abnormalities that may indicate underlying heart problems. ^17, 18^

The heart’s electrical activity, generated by the spontaneous depolarization and repolarization of the heart muscle, is detected by electrodes placed on the surface of the body. These electrodes are usually attached to the patient’s limbs and chest. A standard ECG records this activity from 12 different perspectives or leads, each offering a unique view of the heart’s electrical function. ^19^

**Figure 2.**
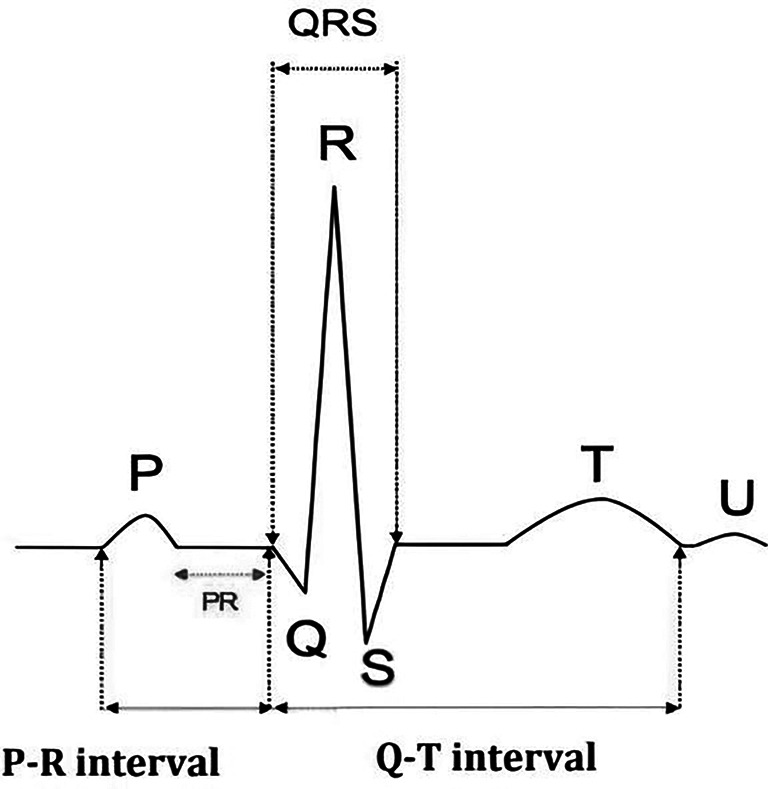
ECG waves and intervals. ^17, 18^

The fundamental components of a typical ECG waveform are illustrated in Figure 2, detailing the various intervals and segments essential for cardiac analysis. ^19^

○ P Wave: The P wave represents atrial depolarization, which is the electrical activation of the atria. This occurs just before the atria contract, pushing blood into the ventricles. The P wave is generally smooth and rounded, and any deviation from this shape may indicate atrial abnormalities such as atrial hypertrophy or atrial fibrillation.
○ QRS complex: The QRS complex represents the rapid depolarization of the right and left ventricles, necessary for the heart’s main pumping action. The QRS complex is usually sharp and narrow, indicating that the ventricles are depolarizing rapidly and efficiently. The duration, amplitude and morphology of the QRS complex are essential to the diagnosis of various conditions, such as ventricular hypertrophy, bundle branch blocks and myocardial infarction.
○ T wave: The T wave displays the ventricular repolarization phase when the ventricles rest before the next heartbeat. This phase is crucial, as it allows the heart muscle to recover and prepare for the next depolarization cycle. The T wave should be vertical in most leads and is usually asymmetrical. T-wave abnormalities, such as inversion or flattening, may indicate ischemia, electrolyte imbalances or other pathological conditions.

### B. Clinical applications of ECG

The ECG is essential for the diagnosis and management of a wide range of cardiac pathologies ^20^:

○ Arrhythmias: The ECG can detect abnormal heart rhythms, such as atrial fibrillation, atrial flutter and ventricular tachycardia, which may be symptomatic or asymptomatic.
○ Myocardial infarction: ECG changes may indicate an ongoing or past myocardial infarction. Specific patterns, such as ST-segment elevation or pathological Q waves, are critical markers.
○ Electrolyte imbalances: hyperkalemia and hypokalemia can produce characteristic ECG changes, facilitating the diagnosis and management of these conditions.
○ Structural abnormalities: Heart chamber enlargement and other structural heart diseases can be deduced from specific ECG changes.

### C. Conventional ECG system

**Figure 3.**
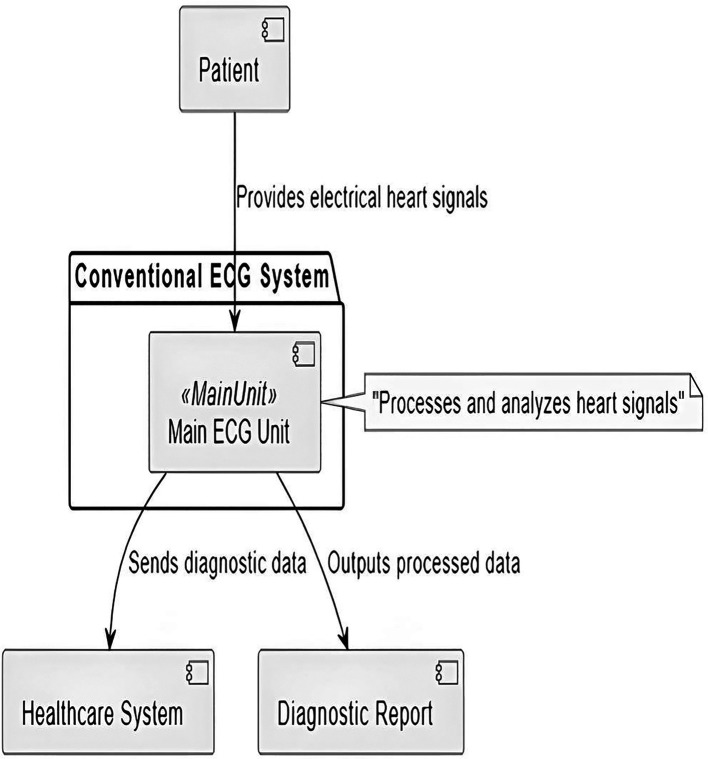
Overview of a conventional ECG system.

To provide a comprehensive understanding of the components and interactions within a conventional ECG system, Figure 3 offers a clear and structured visualization. It highlights the flow of data and the roles of each component in the process ^21^:

○ Patient: the source of the cardiac electrical signals essential to ECG analysis. This is where the diagnostic process begins.
○ Main ECG unit: acts as the system’s central processing unit. It receives the patient’s cardiac signals and processes them to extract meaningful data. This unit is responsible for initial reception and preliminary processing, which includes noise reduction and signal amplification to ensure data clarity and accuracy.
○ Healthcare system: receives processed diagnostic data from the ECG unit. These are external systems (such as hospital information systems or other medical record systems) that analyze the data in greater detail, store it for future reference or use it for immediate medical action. This connection emphasizes the integration of the ECG system with wider healthcare management systems.
○ Diagnostic report: This is the output from the ECG system, where the processed data is formatted into a diagnostic report. The report usually includes interpretations of the ECG results, which are crucial for clinical decision-making. Reports can be printed, displayed on screens or transmitted electronically to other parts of the healthcare system.

### D. ECG artifacts and their impact on ai-based analysis

One of the main challenges associated with interpreting ECG signals, particularly in the context of AI-based analysis, is the presence of artifacts. These artifacts are distortions that mask or alter the actual cardiac signals.

They can be classified as physiological artifacts (e.g., muscle tremors, breathing) and non-physiological artifacts (e.g., electrode movement, poor contact, baseline drift, and interference from power lines). In conventional clinical settings, expert cardiologists can often identify and ignore these distortions. However, in AI-based ECG analysis, where models rely on the fidelity of input data, artifacts pose a significant risk of false arrhythmia detection, classification error, and overfitting on irrelevant features. ^22, 23^

Many studies have shown the sensitivity of ECG processing algorithms to these types of noise. For example, Gupta et al. ^22^ demonstrated that even advanced methods such as chaos-based R-peak detection can be significantly affected by signal corruption. Similarly, Huque et al. ^24^ reported that HMM-based supervised learning frameworks are particularly susceptible to distorted input signals. The study by Sahoo et al. ^23^ also highlighted that most machine learning models require significant preprocessing or exhibit reduced reliability when exposed to artifact-laden ECG data.

To solve this problem, researchers have developed various signal denoising and preprocessing techniques. For example, wavelet transforms and principal component analysis (PCA) have been used to clean ECG signals and extract relevant features in noisy environments. ^24, 25^ These approaches aim to reduce the influence of high-frequency noise or baseline drift without compromising waveform features important for diagnosis. Gupta et al. ^26^ applied an FrWT-based arrhythmia detection method enhanced by PCA and demonstrated improved robustness in real-world conditions.

## IV. Analysis

### A. Evolution of publication years

1. **Annual trends in article publications**

**Figure 4.**
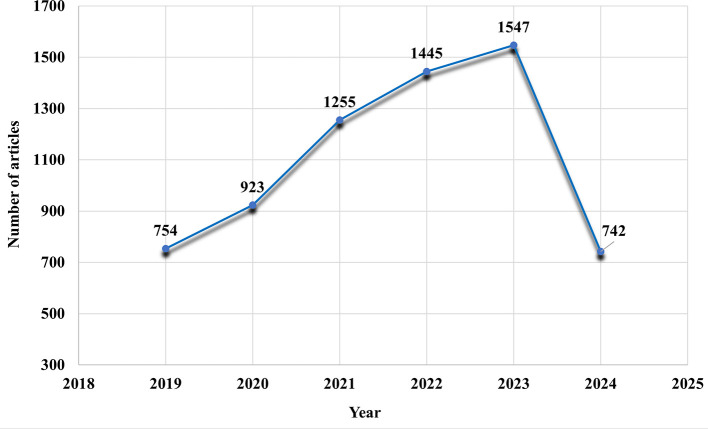
Annual scientific production.

Annual scientific output in the field of ECG devices and AI in recent years shows a significant upward trend, accelerating in particular during the COVID-19 pandemic ( Figure 4).

Initially, publication growth between 2019 and 2020 was steady, with 754 articles published in 2019. An increase is observed from 2020 onwards, with publications rising from 754 in 2019 to 923 in 2020, and significantly to 1255 in 2021. This increase is attributed to the influence of the pandemic, which demonstrated the need for advanced remote diagnostic systems in the face of restricted access to healthcare and the increased burden on medical facilities.

The global health crisis underscored the need for advanced, non-invasive monitoring technologies, such as AI-enhanced ECG devices, which attracted growing research interest as the medical community sought new ways to manage patient care remotely. This increase reflects the urgent need to develop reliable, scalable solutions in a context of restrictions on in-person medical visits and increased burden on healthcare systems.

In 2022, the number of articles continued to rise, reaching 1445 publications, then 1547 in 2023, confirming the sustained interest and ongoing development in this field, driven by the persistent challenges posed by COVID-19 and the recognition of potential future pandemics. However, in 2024, a significant drop in publications to 742 is recorded. This reduction could be explained by the fact that we are probably still at the beginning of 2024, which means that data for this year are incomplete. Other factors could also explain this drop. It’s possible that the intense interest generated by the pandemic is reaching a saturation point, with many solutions and innovations already in place. Researchers can now turn their attention to new challenges or further develop technologies already developed, rather than initiating new publications. This trend underlines the importance of considering the temporal and contextual aspects of publications when analyzing research trends, especially in dynamic fields such as medical technology and artificial intelligence.

2. **Most impactful documents**

Using biblioshiny, a list of the most globally cited documents was constructed based on total citations as presented by Table 1. The first-ranked paper is by Attia ZI, published in 2019, which has received a total of 744 citations, averaging 124 citations per year, and has a normalized citation count of 24.20. This work uses an AI-based ECG algorithm to identify patients with atrial fibrillation. ^27^ This article demonstrates how AI improves cardiovascular diagnostics and helps with better patient management. Following this, Huang J’s paper (2019), ranks second with 384 total citations, an average of 64 citations per year, and a normalized citation count of 12.49. ^28^ The research uses neural networks for the classification of arrhythmias and myocardial infarction. The third-ranked paper by Baloglu UB, published in 2019 with 314 total citations, 52.33 citations per year on average, and a normalized count of 10.21. ^29^ The research demonstrates the importance of deep learning techniques for improving the accuracy of ECG diagnoses. On the fourth position is Andersen RS’s paper, published in 2019 with 286 total citations, 47.67 citations per year, and a normalized citation count of 9.30. ^30^ Their document shows the importance of rapid diagnostics for cardiovascular disease management. Finally, Yao Q’s paper, published in 2020, ranks fifth with 281 total citations, averaging 56.20 citations per year, and a normalized citation count of 11.83. ^31^ Their research uses deep learning for real-time arrhythmia detection showing the importance of rapid diagnostics for cardiovascular disease management.

**Table 1.** Top 5 must globally cited documents.

Rank	Paper	Total citations	TC per year	Normalized TC
1	ATTIA ZI, 2019, LANCET ^27^	744	124	24,20
2	HUANG J, 2019, IEEE ACCESS ^28^	384	64	12,49
3	BALOGLU UB, 2019, PATTERN RECOGN LETT ^29^	314	52,33	10,21
4	ANDERSEN RS, 2019, EXPERT SYS APP ^30^	286	47,67	9,30
5	YAO Q, 2020, INF FUSION ^31^	281	56,20	11,83

These documents reveal the growing impact of AI in ECG technologies. They show how AI enables more accurate diagnoses, continuous monitoring and opens up new avenues for medical research. AI is emerging as an essential tool for improving cardiovascular healthcare, as evidenced by the numerous citations and lasting influence of this work.

### B. Main research keywords in AI for ECG technology

The analysis of the main keywords in this field also shows several distinct and interconnected clusters. Each represents a key aspect of AI research and application in this field.

**Figure 5.**
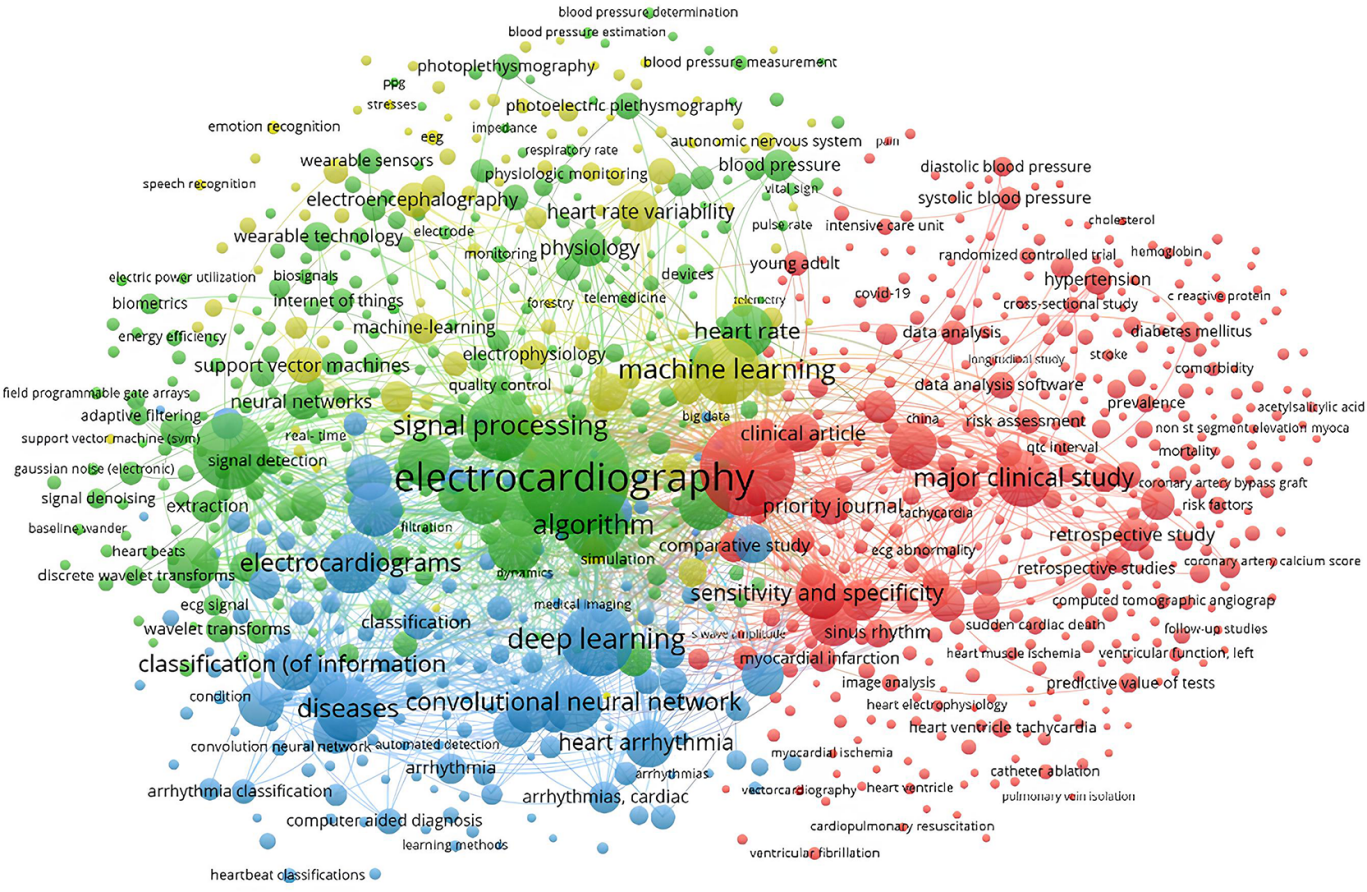
Co-occurence keywords network.

The green cluster in Figure 5, which focuses on terms such as ‘machine learning’, ‘signal processing’ and ‘neural networks’, highlights the importance of machine learning techniques and signal processing in ECG analysis. Researchers in this cluster are focusing on the development of algorithms to improve the detection and classification of cardiac arrhythmias. They stress the need to ensure that these technologies are robust and applicable in real clinical environments in order to maximize their usefulness and impact.

The blue cluster includes keywords such as “deep learning”, “convolutional neural network” and “arrhythmia classification”. This cluster is strongly focused on the use of deep neural networks and other advanced AI methods. They are also used to diagnose cardiac arrhythmias. The aim is to provide more accurate and faster diagnoses, but the practical application of these technologies still needs to be validated on a large scale to ensure their reliability and effectiveness in various clinical contexts.

The red cluster focused on terms such as ‘major clinical study’, ‘sensitivity and specificity’ and ‘data analysis’. This highlighted the importance of clinical studies and data analysis to validate AI technologies in healthcare. This cluster shows efforts to evaluate the clinical effectiveness of new technologies and their integration into routine medical practices. However, it is important to standardize evaluation methods to ensure the comparability of results between different clinical studies.

The yellow cluster with keywords such as “wearable sensor”, “photoplethysmography” and “heart rate variability” shows the integration of wearable sensors and non-invasive technologies in the monitoring and analysis of ECG signals. Research in this area aims to develop wearable devices capable of continuously monitoring cardiac parameters. Although these technologies are promising, questions persist about their accuracy, patient comfort and ability to be integrated into existing healthcare systems.

Overall, Figure 5 provides a good illustration of the interdisciplinary nature of AI-enabled ECG device research.

However, some areas, such as wearable sensors and machine learning methods are more densely explored than others. This suggests potential areas for future research. The concentration of certain keywords around specific themes may indicate research silos, where teams could benefit from closer interdisciplinary collaboration to fill gaps and strengthen innovation. This keyword analysis provides an overview of current trends and research priorities, while highlighting opportunities for collaboration and future development to improve ECG devices using artificial intelligence.

### C. Author influence and collaboration

Analysis of the influence of authors in the field of ECG devices using AI shows that Acharya UR has the highest h_index of 29. This indicates that his work is widely cited and recognised. He also has the highest number of total citations (2668) and a g_index of 51. This underlines the breadth and impact of his publications. LI Y follows with an h_index of 25 and a g_index of 42. LI Y has published the largest number of articles (127) and has accumulated 2086 citations. This shows his strong research activity and the importance of his contributions. LIU C, with an h_index of 23 and a g_index of 43, also shows notable influence. He has published 79 articles with 1995 citations.

Table 2 ranks the top 10 authors according to their contributions since 2019. The m_indexes show that these researchers maintain a high level of productivity throughout their career. Acharya UR and LI Y’s m_index scores, above 4, reflect remarkable productivity. This trend highlights the growing interest in AI-based healthcare technologies and the significant impact of these authors in the field of ECG devices.

**Table 2.** Authors' impact and productivity metrics in ECG and AI research (2019-2024).

Author	H_index	G_index	M_index	Tc	Np
ACHARYA UR	29	51	4,833	2668	51
LI Y	25	42	4,167	2086	127
LIU C	23	43	3,833	1995	79
WANG H	22	36	3,667	1402	76
FRIEDMAN PA	21	50	3,5	2594	71
NOSEWORTHY PA	20	49	3,333	2430	61
ATTIA ZI	19	48	3,167	2374	63
LIU Y	19	35	3,167	1432	91
WANG Y	19	32	3,167	1210	103
ZHANG H	19	31	3,167	1149	92

**Figure 6.**
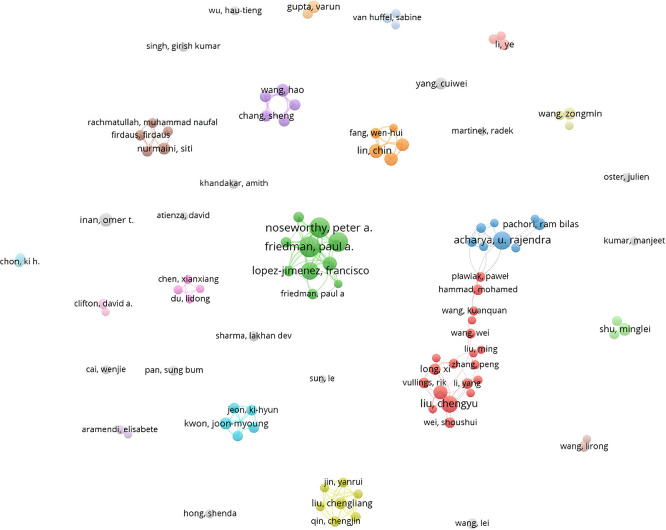
Author collaboration network.

The model of collaboration between these authors in this field shows interesting dynamics and significant trends ( Figure 6).

Firstly, it is clear that certain authors, such as Acharya UR play an important role in the network of collaborations. This type of centralization can be beneficial, as it enables knowledge to be disseminated quickly and research projects to be coordinated effectively. The close collaboration between Acharya UR, Tan RS, and Pachori RB shows strong cooperation, which is often essential for the rapid development of new technologies. However, this centralization can also pose problems, such as over-reliance on a limited number of influential researchers. This can limit the diversity of perspectives and ideas, and potentially hinder innovation in the long term.

In parallel, LI Y and his collaborations with Liu C and Zhang H show significant but less dense connections. This may indicate more specialized research and less diffusion of knowledge between groups. Less dense clusters may be a sign of fragmentation in the field, where research focuses on specific niches without sufficient integration with other subfields. This fragmentation could limit the potential synergies between different approaches and technologies, thus slowing down overall progress.

The cluster around Noseworthy PA and Friedman PA illustrates a well-connected network, but it is questionable whether these collaborations are sufficient to cover all the research needed in this field. Intense collaborations within small groups can lead to significant advances, but they also run the risk of creating knowledge silos, where innovations remain confined to a small group of researchers.

It is also notable that some authors seem isolated or less connected. These authors could represent innovative approaches or important research niches that are not yet integrated into the main network. This raises the question of how these researchers can be better integrated into the collaborative network to maximize their contribution and avoid duplication of effort.

### D. Influential journals

The analysis highlights the main journals contributing to AI-enhanced ECG device research, presenting their performance. IEEE Access stands out with the highest h_index of 44, a g_index of 69 and an m_index of 7,333. It has a total of 6,562 citations from 284 publications. This indicates its substantial impact and wide recognition in the research community. The journal’s high metrics reflect its role as a leading outlet for AI and biomedical engineering research. It provides a platform for the dissemination of influential studies. Biomedical Signal Processing and Control follows with an h_index of 34, a g_index of 55 and an m_index of 5,667, accumulating 5,504 citations from 401 articles. The journal’s focus on signal processing and control systems in biomedical applications making it an essential source of knowledge for advances in AI-based ECG technologies. Its high number of citations and significant number of publications indicates its importance in this niche field. Sensors (Switzerland) also ranks highly, with an h_index of 34, a g_index of 48 and an m_index of 5,667. It received 3,677 citations from 137 articles. This journal plays a central role in the field of sensor technology, particularly in the development of portable, non-invasive monitoring systems, which are an integral part of modern ECG devices.

Overall, Table 3 illustrates the key journals playing in the advancement of AI-enhanced ECG device research. The high citation counts and robust indices of these journals reflect their importance and influence in the academic community. These journals serve as essential platforms for publishing innovative research. This facilitates the dissemination of new ideas and technological advances in ECG devices and AI applications. Measurements suggest that researchers frequently refer to these publications. They underline their relevance and the quality of the research they attract.

**Table 3.** Top 10 journals in AI-enhanced ECG research.

Element	h_index	g_index	m_index	TC	NP
IEEE ACCESS	44	69	7,333	6562	284
BIOMEDICAL SIGNAL PROCESSING AND CONTROL	34	55	5,667	5504	401
SENSORS (SWITZERLAND)	34	48	5,667	3677	137
IEEE JOURNAL OF BIOMEDICAL AND HEALTH INFORMATICS	33	51	5,5	3523	171
COMPUTER METHODS AND PROGRAMS IN BIOMEDICINE	29	48	4,833	2730	109
COMPUTERS IN BIOLOGY AND MEDICINE	29	42	4,833	2175	112
IEEE TRANSACTIONS ON BIOMEDICAL ENGINEERING	24	35	4	1507	83
IEEE TRANSACTIONS ON INSTRUMENTATION AND MEASUREMENT	22	37	3,667	1613	103
IEEE SENSORS JOURNAL	20	30	3,333	1200	91
IEEE TRANSACTIONS ON BIOMEDICAL CIRCUITS AND SYSTEMS	20	35	3,333	1315	65

### E. Institutions and their contributions

1. **Leading institutions**

An analysis of the most active institutions in the publication of articles on ECG devices using AI shows some significant trends ( Table 4). The Mayo Clinic in Rochester in the United States stands out with a high number of articles published. This institution is world-renowned for its expertise in cardiovascular medicine and plays an important role in the development of AI ECG technologies. The participation of several Mayo Clinic departments in the publications reinforces this observation. It also highlights a solid research infrastructure and effective internal collaboration.

**Table 4.** Major institutions in AI-enabled ECG research.

Institutions	Documents	Citations	Total link strength
DEPARTMENT OF CARDIOVASCULAR MEDICINE, MAYO CLINIC, ROCHESTER, MN, UNITED STATES	41	1457	173
DEPARTMENT OF ELECTRONICS AND COMPUTER ENGINEERING, NGEE ANN POLYTECHNIC, SINGAPORE	15	1361	159
SCHOOL OF MEDICINE, FACULTY OF HEALTH AND MEDICAL SCIENCES, TAYLOR'S UNIVERSITY, SUBANG JAYA, 47500, MALAYSIA	6	919	144
DUKE-NUS MEDICAL SCHOOL, SINGAPORE	10	888	128
DEPARTMENT OF NEUROLOGY, MAYO CLINIC, ROCHESTER, MN, UNITED STATES	9	921	122

The University of Wuhan in China also shows a notable ability to integrate the physical sciences and technology into medical research. Its School of Physics and Technology is a major contributor, with numerous articles published. This institution is demonstrating the importance of an interdisciplinary approach to progress in the field of ECG devices using AI.

Seoul, South Korea is another example of the growing importance of Asian research. The contribution from this region highlights the effective integration of technological innovations into medicine.

These institutions show how leaders in the field are playing an important role in the development and advancement of AI-enabled ECG technologies. Their collaborative efforts foster rapid innovation and continuous improvement of medical devices.

2. **Collaboration among institutions**

The analysis showed significant dynamics in citation rates between institutions in the field of AI-enabled ECG devices. It highlights the main contributing institutions and their collaborative networks. The Department of Cardiovascular Medicine at the Mayo Clinic in Rochester clearly stands out with 41 papers and a total of 1457 citations, as well as a total link strength of 173. This institution plays a central role, indicating substantial scientific production and significant influence through numerous citations. Similarly, the Department of Electronics and Computer Engineering at Ngee Ann Polytechnic in Singapore, with 15 papers and 1361 citations, and a total link strength of 159, is also a key contributor. This demonstrates the successful integration of electronics and computer engineering in medical device research. The high number of citations for these two institutions shows the substantial recognition of their work by other researchers.

The School of Medicine, Faculty of Health and Medical Sciences, despite publishing only 6 papers, receives 919 citations and has a total link strength of 144. These figures show that despite a smaller number of publications, the research carried out by this institution is of high quality and has a significant impact.

**Figure 7.**
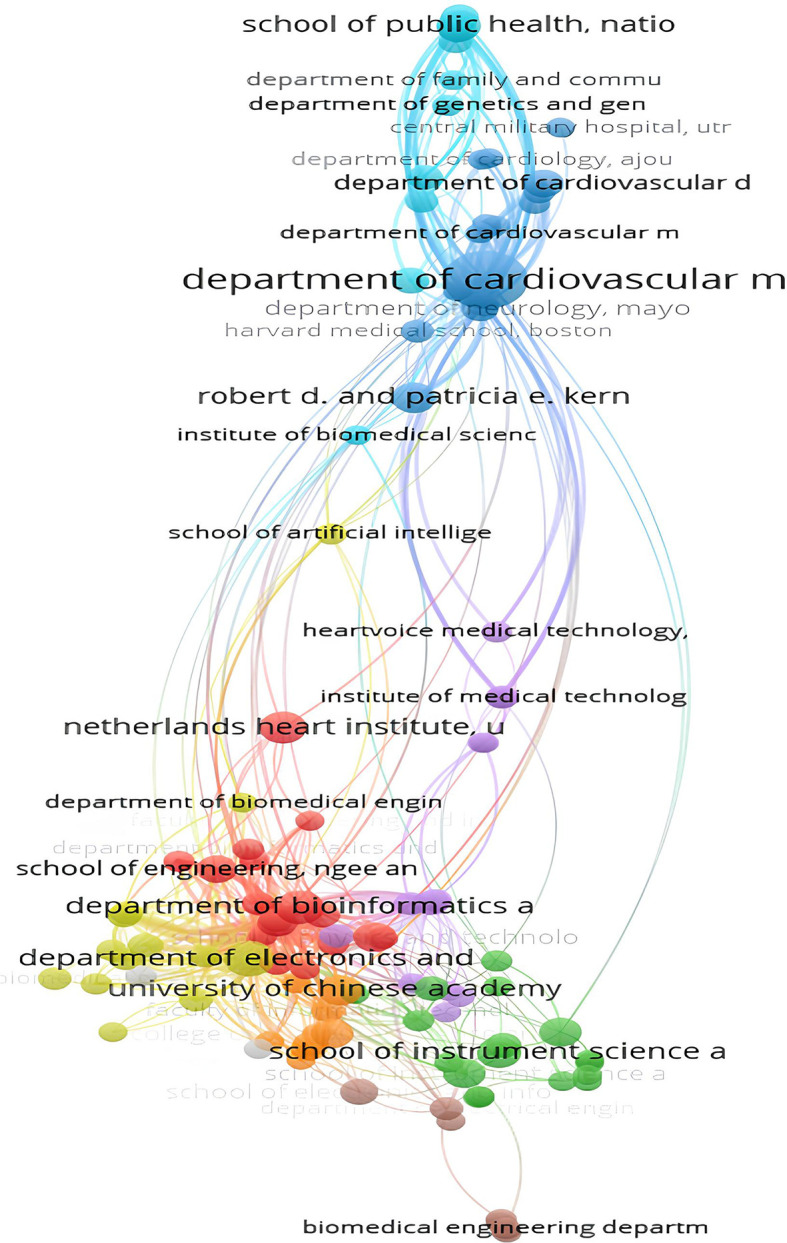
Collaboration network among top institutions in AI-enhanced ECG research.

Figure 7 provides a visual illustration of the citations between these institutions. It highlights the centrality of certain organizations. The Mayo Clinic’s Department of Cardiovascular Medicine is a central node with numerous links, indicating frequent recognition and collaboration by other researchers. The dense clusters around the Mayo Clinic and Singapore institutions such as Duke-NUS Medical School and the National Heart Centre Singapore show close cooperation and research synergy that facilitate rapid technological advancements.

However, some institutions appear isolated on the network map, such as the Department of Biomedical Engineering. This isolation may indicate specialization in very specific niches or less collaboration with other institutions.

### F. Countries and their contributions

1. **Principal countries in research contributions**

Analysis of the table of countries of corresponding authors in the field of ECG devices using AI highlights varied dynamics in terms of scientific production and international collaboration. China stands out with 1,572 articles, demonstrating its significant scientific output. It has 1256 single-signature papers (SCP) and 316 multiple-collaboration papers (MCP), the MCP ratio of 0.201 indicates a moderate tendency to collaborate with researchers from other countries. Although China produces a large number of articles, a significant proportion of this production is carried out independently.

India follows with 899 articles. Its MCP ratio of 0.097, the lowest among the countries listed, suggests that most of the research is carried out independently, without any significant international collaboration. This low level of collaboration could limit the impact and dissemination of Indian research at global level.

The United States comes third with 643 articles. Their MCP ratio of 0.247, one of the highest, shows a strong inclination to collaborate internationally. This reflects the globalized nature of American research and its integration into world research networks, favoring greater dissemination and a more significant impact of their work.

Overall, Table 5 reveals significant disparities in international collaboration rates. Countries such as the United States and the United Kingdom, with high MCP ratios, show a strong inclination to collaborate beyond their borders, which can amplify the impact of their research. On the other hand, countries such as India and China, despite their high output, show less marked tendencies towards international collaboration, which could limit the dissemination and overall impact of their work. This trend could be explained by cultural factors, research infrastructures or national policies on scientific collaboration.

2. **International collaborative networks**

**Table 5.** Leading countries in ECG and AI research.

Country	Articles	SCP	MCP	Freq	MCP_Ratio
CHINA	1572	1256	316	0,236	0,201
INDIA	899	812	87	0,135	0,097
USA	643	484	159	0,096	0,247
KOREA	306	248	58	0,046	0,19
IRAN	168	141	27	0,025	0,161
ITALY	177	128	49	0,027	0,277
JAPAN	141	123	18	0,021	0,128
GERMANY	142	91	51	0,021	0,359
UNITED KINGDOM	162	76	86	0,024	0,531
CANADA	108	75	33	0,016	0,306

Another important aspect is collaboration between countries in this field. The analysis shows several potential clusters and interesting collaborative dynamics. The cluster comprising the United States, Canada and several European countries is central. It is characterized by strong transatlantic cooperation. The United States emerges as a central node with numerous connections to other countries, notably Canada, the United Kingdom, Germany, Italy and Belgium. This North American and European collaboration reflects a high level of integration and scientific cooperation which promotes shared innovation. European countries such as Italy, Spain and Belgium are strongly interconnected and form a sub-cluster within this larger entity. This illustrates a solid infrastructure for scientific collaboration within Europe and with North America.

The Asian cluster of China, India, Taiwan and Japan shows close regional collaboration and significant interaction between these countries. China and India, with large node sizes, are leaders in terms of scientific production in this field, but their collaborations seem to be more regional than international. Taiwan stands out for its significant connections with other Asian countries, while Japan, although important, seems less centralized than China and India. This cluster highlights the importance of intra-regional collaboration in Asia.

The Eastern Europe and Middle East cluster, which includes Iran and Turkey. This cluster shows collaboration with European and Asian countries. Turkey, in particular, acts as a bridge between Europe and Asia, encouraging scientific exchanges between these regions. Overall, the diagram illustrates a high density of international collaborations, with countries such as the United States, China and India playing central roles. The United States shows a strong tendency to collaborate with Europe and Canada, while China and India focus more on regional collaborations in Asia.

**Figure 8.**
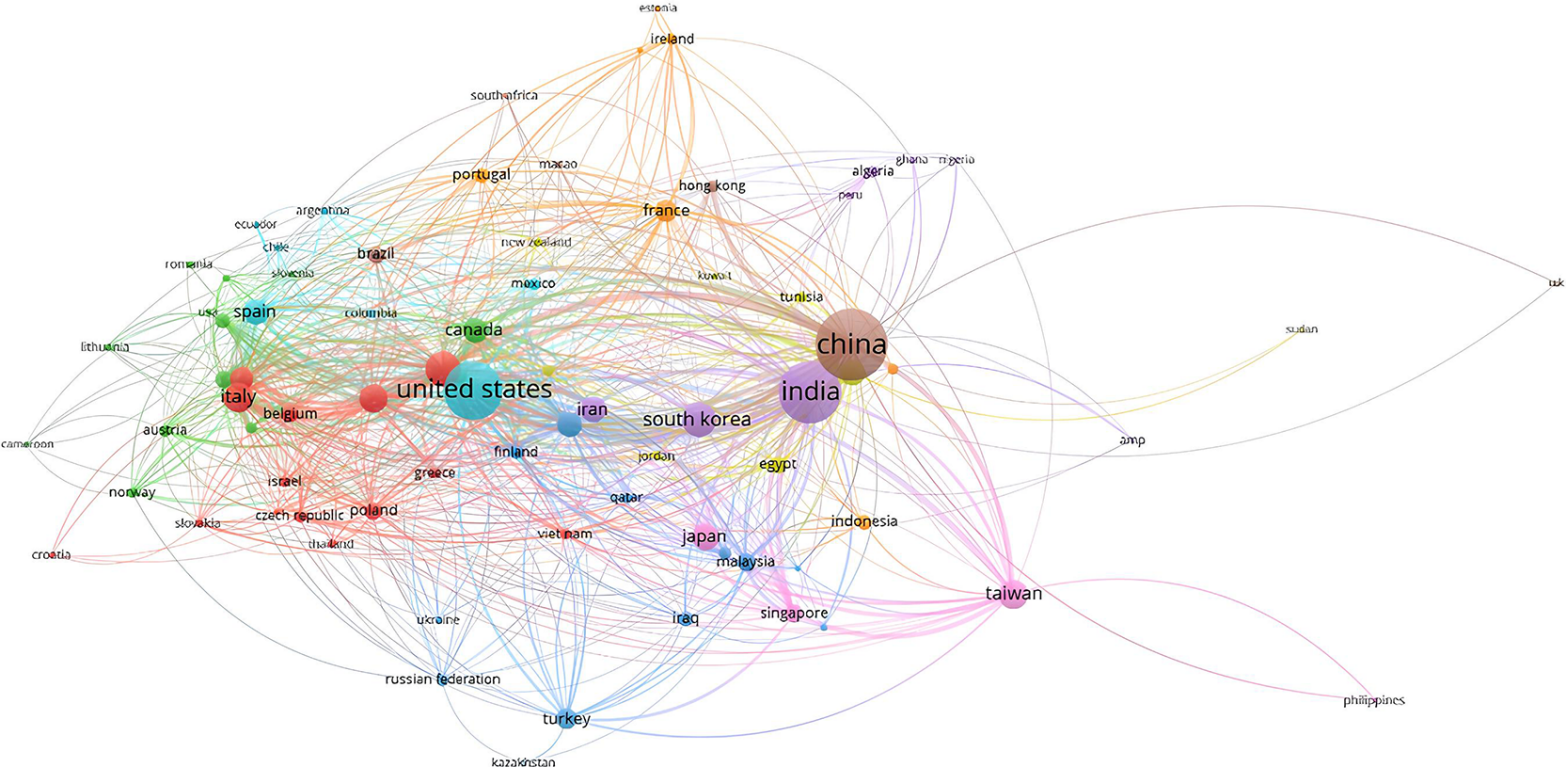
Country collaboration network.

However, there is also relative isolation, particularly in some African and South American countries, suggesting less integration into the global research network. To maximize the overall impact of research into AI-enabled ECG devices, it would be beneficial to encourage more inclusive collaborations that integrate these less connected regions. Figure 8 shows a complex but highly interconnected collaboration dynamic, with strong regional clusters and a notable ability to exchange knowledge across borders. Strengthening international collaborations could further improve innovation and the dissemination of advanced technologies in the field of AI-based ECG devices.

## V. Discussion

### A. Africa’s position in electrocardiogram research

The analysis shows a significant under-representation of African countries in AI-enhanced ECG research. It highlights the critical challenges and obstacles facing African scientists in this rapidly evolving field. African researchers and institutions have minimal visibility in the global AI-enhanced ECG research field. This is marked by the absence of African countries and institutions among the main contributors in terms of publications, citations and collaborative networks. Leading institutions from the USA, Europe and Asia dominate the field without significant representation from Africa. The study by Tuyishime et al. (2022) ^32^ shows that publications from African institutions account for less than 1% of articles published in the most influential cardiology journals.

One of the main obstacles is the limited research infrastructure in many African countries. The advanced facilities needed to conduct high-quality research in AI and medical technology are often unavailable. This strongly limits the ability of African researchers to engage in advanced technology related studies. Balogun et al (2023) ^33^ noted the absence of laboratories equipped with advanced technologies in several African countries. This affects research conducted in Africa as compared to research conducted in developed countries. In addition, AI and medical device research generally requires substantial financial investment which is the main challenge for Africa. This financial constraint limits the scope and scale of research activities that Africa can conduct. Overland et al. (2021) ^34^ analyzed funding flows and found that grants dedicated to research in Africa are largely insufficient compared to those awarded to European and American institutions. The cost and maintenance of AI and ECG technology also present significant obstacles, particularly for widespread adoption and research in resource-limited settings. For example, Bodagh et al. (2023) ^35^ pointed out that advanced ECG devices require regular updates and costly maintenance. This represents a significant barrier for African institutions.

The lack of strong collaborative networks further isolates African researchers. Countries such as the United States and the United Kingdom benefit from high levels of international collaboration. This improves the impact and dissemination of their research. Townsend et al. (2023) ^36^ have shown that American researchers frequently publish with international co-authors, thus improving the reach and impact of their work. In contrast, African researchers often have fewer opportunities to collaborate with leading global institutions. This limits their exposure to new methodologies, technologies and ideas essential to advancing research and innovation. Jackson et al. (2022) ^37^ found that African researchers rarely participate in international collaborative research projects.

Efforts to foster collaborations are essential which necessitates international partnerships and research consortia. This will result into integration of African researchers into the global scientific community. For example, Omosa- Manyonyi et al. (2023) ^38^ described a regional training program in East Africa that significantly improved the skills of local researchers and facilitated international collaborations.

The under-representation of African researchers has important implications. It limits the diversity of perspectives and approaches needed to address global health challenges globally. Diverse research teams bring varied experiences and ideas that can lead to more innovative and inclusive solutions. The absence of African voices means that the specific health challenges and needs of African populations may not be sufficiently addressed. Farrell et al. (2023) ^39^ also noted that healthcare technologies developed without taking local particularities into account often perform less well when applied in African contexts.

This situation perpetuates a cycle of underdevelopment in scientific research on the continent. Without substantial contributions to high-impact research areas, African countries struggle to attract funding, build advanced research facilities and develop the human capital needed to compete on a global scale. This gap limits not only scientific progress, but also the potential health benefits that could grow from inclusive research efforts. Yankam et al. (2023) ^40^ have shown that the lack of research funding and infrastructure prevents African researchers from fully participating in international projects. Building local research capacity and investing in education and training programs are essential steps to breaking this cycle and empowering African researchers. Asubiaro & Shaik (2021) ^41^ have suggested that specific training programs and investment in research infrastructure are essential to improve the participation of African researchers in advanced research projects.

Efforts to close this gap are essential. The creation of AI centers of excellence for global health equity in PRFIs, including African countries has been proposed as a strategic vision to strengthen local research capabilities and address these disparities. Bédair et al. (2023) ^42^ proposed the establishment of regional centers of excellence to stimulate local research and foster international collaborations. Google has also committed to investing heavily in Africa’s digital transformation, including establishing a product development center in Nairobi and expanding its AI residency programs across the continent. ^43^ These initiatives are designed to improve research infrastructures, provide financial support and foster international collaborations, ultimately aiming to integrate African researchers more fully into the global scientific community.

## VI. Limitations

This study has a number of limitations that need to be taken into consideration. Firstly, the use of the Scopus database may result in incomplete coverage of relevant publications. This may exclude important searches available in other databases such as PubMed and Web of science. This limitation means that some influential studies may not have been included, potentially skewing the overall analysis.

In addition, the review provides a quantitative view of the research area of ECG devices field, but may not fully capture the qualitative impact and practical implications of AI-enhanced ECG devices in clinical practice. For example, the actual effectiveness, patient outcomes and integration of these technologies into healthcare systems are not discussed in depth. This limits the understanding of their practical value.

The analysis period from 2019 to 2024, while reflecting recent trends and developments, may overlook earlier seminal work that paved the way for current advancements. Important contributions made before 2019 could provide valuable context and highlight the evolution of AI applications in ECG technology.

Furthermore, while the study highlights the significant under-representation of African contributions to research, it does not explore in depth the underlying socio-economic and political factors that contribute to this disparity. Factors such as funding limitations, training opportunities, infrastructure challenges and political environments are essential to understanding the scale of the obstacles facing African researchers. This omission limits the depth of analysis regarding the systemic issues that need to be addressed to foster a more inclusive research environment.

Moreover, the rapid evolution of AI and medical technology means that some discoveries could quickly become obsolete. The AI field is characterized by rapid innovation and continuous improvement, requiring regular updates and follow-up studies to ensure that analysis remains relevant and accurate over time.

Despite its limitations, this study offers important insights that can be applied across various domains. Firstly, by mapping publication trends, institutional output, and collaborations in the field of AI-enabled ECG devices, the findings can serve as a strategic tool for research funders and policymakers to identify underrepresented regions, particularly in Africa, and prioritize equitable investment in digital health technologies. Secondly, it can guide academic institutions and medical training programs in Africa to align their partnerships with emerging trends. This promotes capacity building in AI applications for cardiology. In addition, AI developers and medical device manufacturers can also use these results to target deployment and co-development in low- and middle-income countries where innovation is underdeveloped.

## VII. Conclusion

This study demonstrates the application of artificial intelligence (AI) in ECG devices. Analysis of data from the Scopus database revealed a significant increase in search results compared with 2019, particularly during the COVID-19 pandemic. This underlines the growing importance of AI in this field. Key contributors include researchers such as Acharya UR and LI Y, and institutions such as the Mayo Clinic and Ngee Ann Polytechnic, which have played a central role in advancing AI-based ECG technologies. ^44^ The study also identified distinct clusters in the keyword analysis, focusing on critical aspects of machine learning and signal processing in ECG research. Despite the high results from countries such as China and India, their lower rates of international collaboration could limit the global impact of their research. Overall, this study provides valuable insights into the dynamic and rapidly evolving field of AI applications in ECG devices.

However, the study also highlights the under-representation of African countries in this field of research. Limited infrastructure, financial constraints and fewer opportunities for international collaboration may contribute to this disparity, hence the need for more inclusive research efforts incorporating contributions from under-represented regions such as Africa.

### Ethics and consent

The authors declare that all ethical guidelines were followed in conducting this research.

## Data Availability

Figshare: Metadata extracted from the Scopus database, used to study the countries, authors, organizations and keywords with the greatest impact on the development of AI-enabled ECG devices: https://doi.org/10.6084/m9. figshare.26405617.v1 Data are available under the terms of the Creative Commons Attribution 4.0 International license (CC-BY 4.0).

## References

[1] Kent B James A : Experiences of health professionals towards using mobile electrocardiogram (ECG) technology: A qualitative systematic review *J. Clin. Nurs.* 2023;32:3205–3218. 10.1111/jocn.16434 35765173

[2] Shcheglov B Konoreva N Koval V : The contemporary ways of introduction ECG technology: ML, telemetry and bioauthentifi cation. *Bratisl. Med. J.* 2023;124(10):783–792. 10.4149/BLL_2023_121 37789797

[3] Bouzid Z Al-Zaiti SS Bond R : Remote and Wearable ECG Devices with Diagnostic Abilities in Adults: A State-of-the-Science Scoping Review. *Heart Rhythm.* Jul. 2022;19(7):1192–1201. 10.1016/J.HRTHM.2022.02.030 35276320 PMC9250606

[4] Tuboly G Kozmann G Kiss O : NC-ND license Atrial fibrillation detection with and without atrial activity analysis using lead-I mobile ECG technology. *Biomed. Signal Process. Control.* 2021;66:102462–108094. 10.1016/j.bspc.2021.102462

[5] Kamga P Mostafa R Zafar S : The Use of Wearable ECG Devices in the Clinical Setting: a Review. *Curr. Emerg. Hosp. Med. Rep.* 2022;10(3):67–72. 10.1007/s40138-022-00248-x 35789964 PMC9244148

[6] Zhu J : A Double- electrodes Amplifier use for Portable ECG Equipment. *Highl. Sci. Eng. Technol.* Feb. 2023;32:279–288. 10.54097/HSET.V32I.5179

[7] Chu H Yang C Xing Y : A Portable ECG Patch Monitor Based on Flexible Non-hydrogel Electrode. *J. Med. Biol. Eng.* Jun. 2022;42(3):364–373. 10.1007/S40846-022-00709-4/METRICS

[8] Strik M : The use of smartwatch electrocardiogram beyond arrhythmia detection. *Trends Cardiovasc. Med.* Apr. 2024;34(3):174–180. 10.1016/J.TCM.2022.12.006 36603673

[9] Maurizi N : Can smart devices and AI in cardiology improve clinical practice? *Rev. Med. Suisse.* May 2023;19(828):1041–1046. 10.53738/REVMED.2023.19.828.1041 37222645

[10] Jeppesen J Christensen J Johansen P : Personalized seizure detection using logistic regression machine learning based on wearable ECG-monitoring device. *Seizure.* Apr. 2023;107:155–161. 10.1016/j.seizure.2023.04.012 37068328

[11] Jafari M : Automatic Diagnosis of Myocarditis Disease in Cardiac MRI Modality using Deep Transformers and Explainable Artificial Intelligence. *arXiv.org.* 2022. 10.48550/ARXIV.2210.14611 Reference Source

[12] Laad M Kotecha K Patil K : Cardiac Diagnosis with Machine Learning: A Paradigm Shift in Cardiac Care. *Appl. Artif. Intell.* 2022;36(1). 10.1080/08839514.2022.2031816

[13] Gupta V Mittal M Mittal V : ECG Signal Analysis based on the Spectrogram and Spider Monkey Optimisation Technique. *J. Inst. Eng. India Ser. B.* Feb. 2023;104(1):153–164. 10.1007/S40031-022-00831-6

[14] Indexation Scopus: pourquoi tout ce tapage? - Le Forum Académique International (IAFOR).Accessed: May 19, 2024. Reference Source

[15] Pranckutė R : Web of Science (WoS) and Scopus: The Titans of Bibliographic Information in Today’s Academic World. *Publications.* 2021;9(1):12. 10.1080/08839514.2022.2031816

[16] VOSviewer - Visualisation de paysages scientifiques.Accessed: Aug. 07, 2024. Reference Source

[17] Chamley RR Holdsworth DA Rajappan K : ECG interpretation: Interpretation of the ECG in young, fit, asymptomatic individuals undertaking high-hazard occupations is the topic of the fourth article in the occupational cardiology series. *Eur. Heart J.* Aug. 2019;40(32):2663–2666. 10.1093/EURHEARTJ/EHZ559 31433846

[18] Neri L : Electrocardiogram Monitoring Wearable Devices and Artificial-Intelligence-Enabled Diagnostic Capabilities: A Review. 2023. 10.3390/s23104805 PMC10223364 37430719

[19] Hampton JR Hampton J (Consultant physician) : L’ECG facile.Accessed: Jun. 02, 2024. Reference Source

[20] Rafie N Kashou AH Noseworthy PA : ECG Interpretation: Clinical Relevance, Challenges, and Advances. *Hearts.* Nov. 2021;2(4):505–513. 10.3390/HEARTS2040039

[21] Serhani MA El Kassabi HT Ismail H : ECG Monitoring Systems: Review, Architecture, Processes, and Key Challenges. *Sensors (Basel).* Mar. 2020;20(6). 10.3390/S20061796 32213969 PMC7147367

[22] Gupta V Mittal M Mittal V : R-Peak Detection Using Chaos Analysis in Standard and Real Time ECG Databases. *IRBM.* 2019;40(6):341–354. 10.1016/J.IRBM.2019.10.001

[23] Sahoo S Dash M Behera S : Machine Learning Approach to Detect Cardiac Arrhythmias in ECG Signals: A Survey. *IRBM.* 2020;41(4):185–194. 10.1016/J.IRBM.2019.12.001

[24] Huque ASA Ahmed KI Mukit MA : HMM-based Supervised Machine Learning Framework for the Detection of fECG R-R Peak Locations. *IRBM.* 2019;40(3):157–166. 10.1016/J.IRBM.2019.04.004

[25] Gupta V : Wavelet transform and vector machines as emerging tools for computational medicine. *J. Ambient. Intell. Humaniz. Comput.* 2023;14(4):4595–4605. 10.1007/S12652-023-04582-0/METRICS

[26] Gupta V Mittal M Mittal V : A Novel FrWT Based Arrhythmia Detection in ECG Signal Using YWARA and PCA. *Wirel Pers. Commun.* May 2022;124(2):1229–1246. 10.1007/S11277-021-09403-1/METRICS

[27] Attia ZI : An artificial intelligence-enabled ECG algorithm for the identification of patients with atrial fibrillation during sinus rhythm: a retrospective analysis of outcome prediction. *Lancet.* Sep. 2019;394(10201):861–867. 10.1016/S0140-6736(19)31721-0 31378392

[28] Huang J Chen B Yao B : ECG Arrhythmia Classification Using STFT-Based Spectrogram and Convolutional Neural Network. *IEEE Access.* 2019;7:92871–92880. 10.1109/ACCESS.2019.2928017

[29] Sci-Hub: Classification de l’infarctus du myocarde avec signaux ECG multi-dérivations et CNN profond. Lettres de reconnaissance de formes.Accessed: Jun. 09, 2024. 10.1016/j.patrec.2019.02.016

[30] Andersen RS Peimankar A Puthusserypady S : A deep learning approach for real-time detection of atrial fibrillation. *Expert Syst. Appl.* Jan. 2019;115:465–473. 10.1016/J.ESWA.2018.08.011

[31] Yao Q Wang R Fan X : Multi-class Arrhythmia detection from 12-lead varied-length ECG using Attention-based Time-Incremental Convolutional Neural Network. *Inf. Fusion.* Jan. 2020;53:174–182. 10.1016/J.INFFUS.2019.06.024

[32] Tuyishime H : Authorship Distribution and Under-Representation of Sub-Saharan African Authors in Global Oncology Publications. 2022. Accessed: May 19, 2024. Reference Source 10.1200/GO.22.00020 PMC9225604 35696623

[33] Balogun OD : INTEGRATING AI INTO HEALTH INFORMATICS FOR ENHANCED PUBLIC HEALTH IN AFRICA: A COMPREHENSIVE REVIEW. *Int. Med. Sci. Res. J.* Dec. 2023;3(3):127–144. 10.51594/IMSRJ.V3I3.643

[34] Overland I : Funding flows for climate change research on Africa: where do they come from and where do they go? 2021. 10.1080/17565529.2021.1976609

[35] Bodagh N : GenECG: A synthetic image-based ECG dataset to augment artificial intelligence-enhanced algorithm development. 10.1136/bmjhci-2024-101335 PMC12142132 40451261

[36] Townsend BA Sihlahla I Naidoo M : Mapping the regulatory landscape of AI in healthcare in Africa. *Front. Pharmacol.* 2023;14. 10.3389/FPHAR.2023.1214422 37693916 PMC10484713

[37] Jackson J : Pathways to research leadership for early career researchers in Africa: A potential role for African and Global Funders. *S. Afr. J. High. Educ.* May 2022;36(2):151–172. 10.20853/36-2-4697

[38] Omosa-Manyonyi GS : Establishment and implementation of a regional mucosal training program to facilitate multi-center collaboration in basic and clinical research in Eastern Africa. *F1000Res.* Sep. 2023;12:1243. 10.12688/F1000RESEARCH.138688.1

[39] Farrell AA : Consensus study on factors influencing the academic entrepreneur in a middle-income country’s university enterprise. *J. Entrep. Emerg. Econ.* Aug. 2023;16:1409–1430. 10.1108/JEEE-08-2022-0241

[40] Yankam BM : Task shifting and task sharing in the health sector in sub-Saharan Africa: evidence, success indicators, challenges, and opportunities. *Pan Afr. Med. J.* Sep. 2023;46(11):11. 10.11604/PAMJ.2023.46.11.40984 38035152 PMC10683172

[41] Asubiaro TV Shaik H : Sub-Saharan African Countries COVID-19 Research: An analysis of the External and Internal Contributions, Collaboration Patterns and Funding Sources. *Open Inf. Sci.* Jan. 2021;5(1):263–277. 10.1515/OPIS-2020-0125/PDF

[42] Bedair H : Funding African-led climate initiatives. 10.1038/s41558-023-01670-z

[43] Google Research accélère la croissance de l’IA en Afrique.Accessed: May 19, 2024. Reference Source

[44] Yang M : Knowledge graph analysis and visualization of artificial intelligence applied in electrocardiogram. *Front. Physiol.* Feb. 2023;14:1118360. 10.3389/FPHYS.2023.1118360/BIBTEX 36846320 PMC9947408

