# Supplementary material for: Assessing Africa’s position in the development of AI-enabled ECG devices
Source: F1000Res. 2025 Aug 19;13:1115. Originally published 2024 Oct 2. [Version 2] doi: 10.12688/f1000research.154316.2 (PMC12612617; doi:10.12688/f1000research.154316.2)
Supplement: Peer review history

# Reviewer report: Reviewer response for version 2

**DOI:** 10.5256/f1000research.185967.r428331

**Published:** 2025-11-12

**Authors:** Mouad EL MOUZOUN EL IDRISSI

**Affiliations:** 1 Laboratory of Radiation-Matter and Instrumentation, Hassan First University of Settat, Settat, Morocco; 2 Biomedical Engineering laboratory, Suptech Santé Engineering School, Casablanca, Morocco

The present paper introduces a global analysis of the development of ECG devices, the integration of AI within these technologies, and the impact of Africa's countries in this field.

 Some suggestions:  

 1- You need to present the reason why you have written this paper, in order to show the interest of your work.

 2- you have to present your motivation in the perspectives section to give an idea of the chronology of your work.

 3- Provide a higher resolution of Figures 7 and 8, some words are not clear / incomplete.

 4- Present more technical information of ECG Devices and AI-integrated technologies and compare and discuss their limitations / strengths.

Is the work clearly and accurately presented and does it cite the current literature?

Yes

If applicable, is the statistical analysis and its interpretation appropriate?

I cannot comment. A qualified statistician is required.

Are all the source data underlying the results available to ensure full reproducibility?

Yes

Is the study design appropriate and is the work technically sound?

Yes

Are the conclusions drawn adequately supported by the results?

Yes

Are sufficient details of methods and analysis provided to allow replication by others?

Yes

Reviewer Expertise:

Biomedical Engineering & Artificial Intelligence

I confirm that I have read this submission and believe that I have an appropriate level of expertise to confirm that it is of an acceptable scientific standard.

# Reviewer report: Reviewer response for version 2

**DOI:** 10.5256/f1000research.185967.r406865

**Published:** 2025-08-20

**Authors:** Varun Gupta

**Affiliations:** 1 National Institute of Technology, Ravangla, Sikkim, India

Authors have revised the manuscript very well. Now manuscript may be accepted in current form.

Is the work clearly and accurately presented and does it cite the current literature?

Yes

If applicable, is the statistical analysis and its interpretation appropriate?

Yes

Are all the source data underlying the results available to ensure full reproducibility?

No

Is the study design appropriate and is the work technically sound?

Yes

Are the conclusions drawn adequately supported by the results?

No

Are sufficient details of methods and analysis provided to allow replication by others?

No

Reviewer Expertise:

Biomedical Signal Processing and Control

I confirm that I have read this submission and believe that I have an appropriate level of expertise to confirm that it is of an acceptable scientific standard.

# Reviewer report: Reviewer response for version 1

**DOI:** 10.5256/f1000research.169324.r374888

**Published:** 2025-06-20

**Authors:** Varun Gupta

**Affiliations:** 1 National Institute of Technology, Ravangla, Sikkim, India

1. Explain the main contributions and motivations behind this paper.
2. Explain the computational complexity of the proposed work in the paper.
3. Quality of figures is not up to the mark. Why study is limited to Africa position only.
4. What are the novelties in the proposed work? Is it beneficial for AI enabled ECG signal processing?
5. What are the possible applications of the proposed work?
6. What is the performance evaluating parameters when you are taking part of Africa position?
7. Manuscript needs inclusion of the necessary flow charts and add some more relevant results in the manuscript.
8. Explain the performance evaluating parameters which were considered in the past literature and claim the novelty of the work.
9. Major section on ECG Artifacts is required to enable the AI technique.
10. Some more description on AI techniques/Deep learning techniques like Wasserstein Deep Convolutional Generative Adversarial Network Optimized and Harbor Seal Whiskers Optimization Algorithm are required with its advantages, disadvantages and operating parameters.
11. As paper is based on AI enable ECG devices, following papers may be added-

- Gupta V et al. (2019) - **Ref 1**
- Huque A et al. (2019) - **Ref 2**
- Sahoo S et al. (2020) - **Ref 3**
- Gupta V (2023) - **Ref 4**
- Jung W et al. (2017) - **Ref 5**
- Gupta V et al. (2022) - **Ref 6**

Is the work clearly and accurately presented and does it cite the current literature?

Yes

If applicable, is the statistical analysis and its interpretation appropriate?

Yes

Are all the source data underlying the results available to ensure full reproducibility?

No

Is the study design appropriate and is the work technically sound?

Yes

Are the conclusions drawn adequately supported by the results?

No

Are sufficient details of methods and analysis provided to allow replication by others?

No

Reviewer Expertise:

Biomedical Signal Processing and Control

I confirm that I have read this submission and believe that I have an appropriate level of expertise to confirm that it is of an acceptable scientific standard, however I have significant reservations, as outlined above.

## References

[1] : R-Peak Detection Using Chaos Analysis in Standard and Real Time ECG Databases. *IRBM* .2019;40(6) : 10.1016/j.irbm.2019.10.001 341-354 10.1016/j.irbm.2019.10.001

[2] : HMM-based Supervised Machine Learning Framework for the Detection of fECG R-R Peak Locations. *IRBM* .2019;40(3) : 10.1016/j.irbm.2019.04.004 157-166 10.1016/j.irbm.2019.04.004

[3] : Machine Learning Approach to Detect Cardiac Arrhythmias in ECG Signals: A Survey. *IRBM* .2020;41(4) : 10.1016/j.irbm.2019.12.001 185-194 10.1016/j.irbm.2019.12.001

[4] : Wavelet transform and vector machines as emerging tools for computational medicine. *Journal of Ambient Intelligence and Humanized Computing* .2023;14(4) : 10.1007/s12652-023-04582-0 4595-4605 10.1007/s12652-023-04582-0

[5] : An Arrhythmia Classification Method in Utilizing the Weighted KNN and the Fitness Rule. *IRBM* .2017;38(3) : 10.1016/j.irbm.2017.04.002 138-148 10.1016/j.irbm.2017.04.002

[6] : A Novel FrWT Based Arrhythmia Detection in ECG Signal Using YWARA and PCA. *Wireless Personal Communications* .2022;124(2) : 10.1007/s11277-021-09403-1 1229-1246 10.1007/s11277-021-09403-1

# Response

**Published:** 2025-07-09

**Authors:** Hamza AMEZIANE

**Affiliations:** Universite Hassan II Casablanca Ecole Nationale Superieure d'Arts et Metiers, Casablanca, Grand Casablanca, Morocco

Dear Reviewer Varun Gupta,

 We sincerely thank you for your detailed and constructive feedback on our manuscript. We have carefully considered each of your comments and made the appropriate revisions to improve the quality and clarity of our article. Below is a point-by-point response to your valuable suggestions:

**Comment 1: Explain the main contributions and motivations behind this paper.**

 We clarified the motivation and main contributions in the revised introduction. Specifically, we highlighted that this is the first study that quantitatively assesses Africa's position in AI-based ECG research. The motivation is to address the global disparity in research visibility and collaboration, particularly affecting low- and middle-income countries. This work aims to expose structural inequalities and inspire more inclusive research investment.

**Comment 2: Explain the computational complexity of the proposed work.**

 The present article is a bibliometric study; it doesn't involve algorithm design or system implementation, which would require an analysis of computational complexity. We specified in the methodology section that no algorithmic implementation had been introduced. The study focuses on metadata and publication trends.

**Comment 3: Quality of figures is not up to the mark. Why is the study limited to Africa only?**

 We have significantly improved the quality and clarity of all figures.

 Regarding the geographic focus on Africa, we have now clearly justified this choice in the introduction section. While many studies exist on AI-enabled ECG developments from a technological or clinical perspective, there is a significant lack of literature that evaluates geographical inclusion in this field, especially for low- and middle-income regions like Africa. This focus aims to expose structural imbalances in global research production. It also highlights the disparities in international collaboration networks and encourages equitable participation in AI-driven medical innovation. Our intent is not to exclude other regions but to bring visibility to an underrepresented zone in global health technology discussions.

**Comment 4: What are the novelties in the proposed work? Is it beneficial for AI-enabled ECG signal processing?**

 We clarified the novelty in the abstract and introduction: this is the first paper to quantitatively assess Africa’s involvement in AI-enabled ECG research. While not directly improving signal processing, this study identifies gaps and opportunities for future AI research deployments in low-resource regions.

**Comment 5: What are the possible applications of the proposed work?**

 We thank you for this pertinent observation. While the primary objective of our work was to analyze the global scientific production landscape on AI-based ECG devices, particularly Africa’s contribution, we agree that emphasizing the potential applications strengthens the manuscript's relevance.

 We have therefore clarified this in the revised manuscript by integrating a discussion that highlights how our findings can guide

1. Research funders and policymakers, by offering empirical evidence to support equitable investments in digital health innovation, especially in underrepresented regions;
2. Academic institutions and medical educators, by identifying collaboration networks and publication trends to tailor training and partnership strategies in AI cardiology.
3. Developers of medical AI and device manufacturers, by providing actionable insights to direct innovation, deployment, and co-creation strategies toward low- and middle-income countries, where such technologies can have transformative health impacts.

This addition now reflects the broader practical scope of our study and addresses the application dimension mentioned in the comment.

**Comment 6: What are the performance evaluating parameters when you are taking part of Africa position?**

 We included indicators such as the number of publications, citations per article, h-index, and MCP ratio to assess Africa’s output. These are explained in the Methodology section and used in the Results section for Africa-specific evaluation.

**Comment 9: Major section on ECG Artifacts is required to enable the AI technique.**

 We added a dedicated subsection titled **“D.    ECG Artifacts and Their Impact on AI-Based Analysis”**. It discusses common artifacts and their implications for AI-based ECG interpretation.

**Comment 10: Some more description on AI techniques like WDCGAN and Harbor Seal Whiskers Optimization Algorithm is required.**

 We completely agree with you that a more detailed examination of these technical algorithms is entirely relevant. However, this article is limited to a geographical analysis focused on visibility and collaboration networks, particularly in Africa. Therefore, a detailed comparative study of specific AI architectures is not included in the scope of this paper. We plan to dedicate a specific review article to the technical analysis of AI models in ECG signal processing, including emerging methods such as WDCGAN and metaheuristic optimizations. This study will evaluate their strengths, limitations, and clinical applicability.

**Comment 11: Add the following papers related to AI-enable ECG devices.**

 We thank you for the useful suggestions. All six recommended references (Gupta et al., Huque et al., Sahoo et al., Jung et al.) have been cited in the revised manuscript to strengthen the related work and discussion.

 We believe the revised manuscript now addresses all major concerns and significantly improves the scientific and editorial quality of the work. We truly appreciate your expert insights.

 Sincerely,
